# Investigating the health implications of social policy initiatives at the local level: study design and methods

**DOI:** 10.1186/1471-2458-10-759

**Published:** 2010-12-08

**Authors:** Gemma E Carey

**Affiliations:** 1 Centre for Health & Society, School of Population Health, University of Melbourne, Melbourne, Australia

## Abstract

**Background:**

In this paper we present the research design and methods of a study that seeks to capture local level responses to an Australian national social policy initiative, aimed at reducing inequalities in the social determinants of health.

**Methods/Design:**

The study takes a policy-to-practice approach and combines policy and stakeholder interviewing with a comparative case study analysis of two not-for-profit organisations involved in the delivery of federal government policy.

**Discussion:**

Before the health impacts of broad-scale policies, such as the one described in this study, can be assessed at the population level, we need to understand the implementation process. This is consistent with current thinking in political science and social policy, which has emphasised the importance of investigating how, and if, policies are translated into operational realities.

## Background

Over the last two decades, Australia, like many countries, has experienced widening inequalities in the social determinants of health. From 1996-2007, the former conservative government's policies increased inequality across Australian communities, despite achieving sustained economic growth [1]. Policy analysts argue that this was caused by government action on two levels. Firstly, by pursuing policies, such as disinvestments in public education and healthcare, that actively penalized those at the lower end of the social gradient [2]. Secondly, by contracting out government services to not-for-profit organisations, the government constrained the efforts of non-state actors to address the needs of disadvantaged individuals and communities [3].

In 2007, the Australian Labor Party ran for government on a platform of increased equality and social inclusion. Since taking office they have launched the Social Inclusion Agenda (SIA): an ambitious large-scale social policy initiative designed to re-orientate the delivery of social and public services and redress growing inequality. The government anticipates that the SIA will combat complex and intractable problems of exclusion and disadvantage by promoting social, economic and civic participation and by re-orientating the provision of welfare and social services [4]. Under the government's vision of a socially inclusive Australia, all citizens will have resources and opportunities to: participate in education and training; work in employed, voluntary, family or caring capacities; become engaged in their local communities; and have a voice to effect decisions which influence their lives [4]. While many hope that the SIA will achieve institutional change within government, significant emphasis is being placed on supporting and better facilitating non-state actors to address disadvantage. Primarily, these actors are not-for-profit organisations operating in the welfare sector. This approach is in keeping with international trends; welfare reform is increasingly concerned with altering the way in which welfare systems operate and are organized [5]; to achieve this re-organisation, governments are increasingly looking to the not-for-profit sector [6].

The SIA has the potential to significantly reduce inequalities in the social determinants of health, thereby bringing widespread benefits to individuals and communities. However, given the reliance on non-state actors, the SIA is a challenging and risky policy intervention. The choice of social inclusion as a policy framework adds another layer of complexity to this challenging implementation environment. Individuals who are disadvantaged or marginalised experience multiple, complex, and changing barriers to wellbeing and inclusion [1]. It is important to note that for services to be successful in promoting the inclusion of these individuals, they must be flexible, adaptive and locally responsive [1]. The SIA therefore faces an added challenge of allowing for local flexibility, while enforcing a new guiding principle for welfare and the delivery of services.

To ensure the success of the SIA, significant macro-level implementation is required between different levels, and across different departments, of federal and state governments. The government has also made a strategic commitment to the not-for-profit sector in order to deliver on the outcomes of the SIA. This means that the SIA must also be successfully adapted and implemented at the local level by these organisations. To facilitate this, the government aims to create new partnerships and opportunities to encourage not-for-profit organisations to deliver more innovative, integrated and holistic services for marginalised and disadvantaged people.

The not-for-profit (or third) sector has an important, and increasingly prominent, role in addressing the social determinants of health. Since the 1970s the sector has been an important facilitator of social and civic participation, and is understood to build social capital and promote social cohesion [7]. Research into the social determinants of health has now compiled a considerable body of evidence that links social participation, cohesion and some forms of social capital to health [8,9]. The third sector is also fundamental to more explicitly public health-orientated activities: not-for-profit operations encompass health promotion, community development, community empowerment, and consumer participation [10]. Over the last two decades, the sector has become increasingly involved in the provision of social and public services, which support the health and welfare of the population [3,7,6,11,12]. Not-for-profits are also increasingly seen as important contributors to policies that impact the social determinants of health.

However, as Michael Marmot [13]: p160. argues, "[w]hile the real and potential contribution of the third sector to reducing health inequalities is recognised, there remains concerns about how the sector is supported, both to deliver its services and to effectively engage [with government] as a strategic partner".

The decision to implement the SIA primarily through the not-for-profit sector means that ultimately the success or failure of the policy resides within the organisational contexts of individual not-for-profits. Under the SIA, not-for-profit organisations face a new set of rules - how organisations respond to these rules will influence whether the SIA is realised at the local level [14]. Thus, successful implementation relies upon: the government putting in place the right infrastructure (e.g. funding structures) to allow organisations to address exclusion; the appropriateness of social inclusion as a framework for the sector; and the willingness and capacity of individual not-for-profits to engage with the agenda.

For Australia, the SIA is arguably one of the most significant broad-scale public health policies of recent times. With a focus on redressing inequality, increasing participation and social connectedness and providing better services and support to citizens, it has the potential to make significant inroads into inequalities in the social determinants of health. However, the reliance on the not-for-profit sector raises a number of questions: will there be sufficient change at a state and institutional level to allow not-for-profit organisations to successfully address exclusion? Will organisations respond to the challenges thrown down by the SIA? And, is social inclusion an appropriate, or useful, concept for those who work at the coalface of disadvantage?

## Methods/Design

To answer these questions, this study takes a policy-to-practice approach: examining changes in federal policy through to 'street-level' service delivery. In doing so, it aims to build a practical understanding of how the SIA will impact upon the not-for-profit sector and its ability to address disadvantage and promote social inclusion. The study is funded by the Australian National Health and Medical Research Council and the Sidney Myer Foundation. It received approval from the University of Melbourne School of Population Health Human Ethics Advisory Group.

Core objectives:

1. To determine what changes are occurring under the SIA in the relationships between the not-for-profit sector organisations and other sectors, such as government and private enterprise

2. To examine how this change impacts upon not-for-profit organisations and their ability to promote social inclusion

3. To ascertain if, and how, this change can inform health promotion strategies which seek to address health inequalities through promoting social inclusion

The research takes a policy to practice approach. Case studies of two not-for-profit organisations will be contrasted with the perspectives of policy makers, researchers and other experts in the field. The research will be carried out in three phases:

1. Policy and desktop analysis of government policy and public debate relating to the SIA

2. Ethnographically-informed comparative case study analysis of two not-for-profit organisations involved in the delivery of social services

3. Interviews with experts in the field, such as federal policy makers and not-for-profit researchers

Increasingly, studies of social policy implementation and welfare reform have used inductive case study approaches at the organizational level [15-19]. In the case of social services, this is viewed as a particularly appropriate and productive methodological turn [14,15]. Emerging research in this area highlights the importance of examining whether and how written policies are "translated into operational realities", and the variations and permutations that occur during this process [20]: p145. This 'street-level' analysis addresses current gaps in our understanding of policies and how they work [20]. For example, it gives us a fuller picture of how policies such as the SIA are "produced and experienced in daily life" [20]: p145. Brodkin[20] argues that this approach is most valuable when policy implementation involves "change in organizational practice, discretion by frontline workers, and complex decision-making in a context of formal policy ambiguity and uncertainty" [20]: p145.

Lurie [18] has argued for the use of comparative case study methodologies for street-level analysis of welfare reform. Comparative case studies examine multiple cases within a shared framework, seeking out what is both common and particular, and examines the patterning of variables and relationships [21,22]. However, the use of ethnographic techniques in this area is rare. While ethnography has gained increasing prominence in implementation and welfare studies, it has primarily been used to examine the experiences of clients rather than organizations [23].

### Study protocol

#### Phase one: Desktop & Policy Analysis

A desktop and policy analysis of the SIA will run throughout the duration of the study. Through a descriptive and analytical examination of the development of the SIA and the initiatives which sit under it, this phase will determine: how the SIA is implemented at various levels across and between government(s); how the government is engaging with the not-for-profit sector; and the fit of these activities, and the overall framework of social inclusion, with the sector and the goal of addressing disadvantage.

#### Phase two: Organisational case studies

Phase two uses comparative case study analysis to examine how the SIA impacts upon the not-for-profit sector [24]. While the types of organizations that exist within the third sector are extremely diverse, the experiences of the organizations in this study are likely to give inference to the experiences of other organizations and the sector more broadly [21]. The study will examine organisational culture change and shifts in organisation-government relations under the SIA. In doing so, it will investigate how these changes impact upon the ability of organisations to promote social inclusion. Change may be found in organisational discourses, practices and activities, such as collaborative or innovative approaches to service provision.

Two not-for-profit organisations will be purposefully sampled for comparative case study analysis. A comparative case study methodology has been chosen because the SIA has no clear set of outcomes with regard to its impact on the not-for-profit sector [24]. The project is informed by ethnographic technniques approach and combines semi-structured qualitative interviews with *targeted *participant observation and document analysis [25-28]. This approach enables the two case studies to run in tandem so that time sensitive changes can be captured.

The targeted participant observation will include attendance at events and meetings deemed appropriate for 12 months [28]. In addition, two programs that take a social inclusion approach to service delivery will be selected in each organisation for on-going observation. Up to 10 interviews will be conducted with stakeholders, staff and volunteers (where appropriate). During this time detailed fieldnotes will be kept and interviews will be recorded and transcribed verbatim. A document analysis will also be undertaken at each organisation. This will include documents such as funding agreements, strategic plans, newsletters, and program reports. Analysis will be inductive and thematic.

##### Discussion group

This phase of the study is informed by reciprocal ethnography methodology [25-27]. This stems from feminist research and enables engagement and empowerment of participants, through shared dialogue and the encouragement of shared learning and outcomes [25-27]. This is a socially inclusive methodology in keeping with the study aims. In each not-for-profit organisation a self-selecting discussion group of staff, volunteers and board members will be established. This group will convenea minimum of four times through data collection and analysis to workshop findings and interpretations.

##### Case study sampling

Potential organisations were identified from the Australian Council of Social Services (ACOSS) membership list. ACOSS is the peak not-for-profit organisation for the Australian social services sector. Organisations were selected on the basis of the following criteria combined with a willingness to participate:

• Social services organisation

• Has a service delivery role

• Has an advocacy role

• Has an interest in social justice or social inclusion

• Has a social policy unit.

Of the two organisations included in the study, one advocates for a social inclusion framework for the delivery of social services. This organisation has substantial networks with government. The other organisation has a more arms length relationship with government, and is more reticent about the SIA and the framework of social inclusion for the delivery of services.

#### Phase 3: Stakeholder & policy maker interviews

The last phase of the study will take what has been learnt through the first two phases and contrast it with the perspectives of experts in the field. Federal policy makers, researchers and other experts involved in not-for-profit sector-related research or policy will be interviewed. Areas for inquiry will be determined by the findings from phases one and two and will seek to contrast the experiences of the selected organisations with the perspectives of policy makers and experts.

Criterion-based, purposive sampling of up to 15 individuals chosen on the basis of current/past role in not-for-profit sector-related policy or research will be conducted [29]. Snowball sampling will be carried out - participants will be asked to nominate other stakeholders, until saturation is reached. Interviews will be individual and semi-structured [29,30]. Analysis will be thematic; findings will be contrasted with themes identified in the first two phases of the study.

## Discussion

The evidence on the social determinants of health has meant that public health practitioners are increasingly expected to operate outside the health sector and in partnership with other sectors. The importance of social policy to health therefore cannot be underestimated. However, before the impact of broad-scale social or public health policies such as the SIA can be assessed at the population level, we need to understand how they are produced and experienced in daily-life. To do this, studies must investigate the implementation process. This is consistent with methodological advances occurring in political science and social policy, which have emphasised the importance of investigating how, and if, policies are translated into operational realities.

## List of abbreviations

SIA: Social Inclusion Agenda. ACOSS: Australian Council of Social Services.

## Competing interests

The authors declare that they have no competing interests.

## Pre-publication history

The pre-publication history for this paper can be accessed here:

http://www.biomedcentral.com/1471-2458/10/759/prepub
